# Coffee consumption is positively related to insulin secretion in the Shanghai High-Risk Diabetic Screen (SHiDS) Study

**DOI:** 10.1186/s12986-018-0321-8

**Published:** 2018-11-27

**Authors:** Fei Gao, Yinan Zhang, Sheng Ge, Huijuan Lu, Ruihua Chen, Pingyan Fang, Yixie Shen, Congrong Wang, Weiping Jia

**Affiliations:** 1 Department of Endocrinology and Metabolism, Shanghai Jiao Tong University Affiliated Sixth People’s Hospital, Shanghai Clinical Medical Center of Diabetes, Shanghai Key Clinical Center of Diabetes, Shanghai Diabetes Institute, Shanghai Key Laboratory of Diabetes, Shanghai, China; 2 Shanghai Jiao Tong University Affiliated Sixth People’s Hospital, Shanghai Key Laboratory of Diabetes, The Metabolic Diseases Biobank, Center for Translational Medicine, Shanghai, China; 3 Department of Clinical Nutrition, Shanghai Jiao Tong University Affiliated Sixth People’s Hospital, Shanghai, China; 4 Department of Endocrinology and Metabolism, Shanghai Jiao Tong University Affiliated Sixth People’s Hospital, Shanghai Key Laboratory of Diabetes, The Metabolic Diseases Biobank, Shanghai, China

**Keywords:** Coffee, Diabetes, Insulin secretion

## Abstract

**Background:**

It has been proved that coffee consumption was associated with a lower risk of type 2 diabetes mellitus. But the benefit effect of coffee on hyperglycemia in Chinese population was largely unknown. Besides, the relationship of coffee intake and diabetic pathogenesis was still unclear.

**Methods:**

The study population was selected from the Shanghai High-Risk Diabetic Screen (SHiDS) project. A total of 1328 individuals over 18 years of age who have the information of coffee intake were enrolled in the study from 2012 to 2016. Each participant finished a five-point 75 g oral glucose tolerance test and finished a standard questionnaire. Insulin resistance was evaluated by HOMA-IR and insulin secretion was evaluated by HOMA-β, Stumvoll first phase and second phase indexes.

**Results:**

Coffee consumption group had lower plasma glucose levels at 2-h and 3-h and higher insulin levels at fasting, 30-min and 1-h during OGTT after adjustment with age, fat%, BMI, waist, tea intake, smoking habit, alcohol intake, diabetes family history and educational status (P for PG2h = 0.002; P for PG3h = 0.010; P for FIN = 0.010; P for IN30min = 0.001; P for IN1h = 0.002). Both HOMA-β and Stumvoll formula indexes were positively related to coffee consumption (P for HOMA-β = 0.033; P for Stumvoll first phase = 0.003; P for Stumvoll second phase = 0.001). Logistic regression analysis further confirmed that coffee intake was independently associated with higher levels of HOMA-β and Stumvoll insulin secretion indexes [OR (95% CI) for HOMA-β = 2.270 (1.456–3.538); OR (95% CI) for Stumvoll first phase = 2.071 (1.352–3.173); OR (95% CI) for Stumvoll second phase = 1.914 (1.260–2.906)].

**Conclusions:**

Coffee intake is independently and positively related to pancreatic beta cell function in a large high-risk diabetic Chinese population.

## Background

Coffee is one of the most widely used beverages worldwide. In the recent decades, there are lots of studies about the relationship of coffee consumption and a series of diseases, such as cardiovascular disease [1], cerebrovascular disease [2], insomnia [3] and diabetes [4]. Several meta-analyses confirmed that coffee consumption was associated with a lower risk of type 2 diabetes mellitus [5–7]. As we know, insulin resistance and beta cell function deterioration are the two critical underlying traits of diabetes pathogenesis. However, the connection between coffee and diabetes pathogenesis is still controversial. For instance, a Swedish study implied that coffee intake might involve both improved insulin sensitivity and enhanced insulin response [8]. Nonetheless, some others showed that coffee was associated with insulin sensitivity but not insulin secretion. Arnlov J et al. only found relationship of coffee intake with insulin sensitivity but not with early phase insulin secretion during an oral glucose tolerance test [9]. Thus, it is still significant to further investigate the relationship between coffee and the pathogenesis of diabetes.

China is a newly coffee consumption country, more and more people custom to having coffee frequently. Meanwhile, China has large diabetes epidemic.The estimated overall prevalence of total diabetes and of pre-diabetes is 10.9 and 35.7%, respectively [10]. However, the association between coffee consumption and diabetes or the pathogenesis in Chinese population is largely unknown.

In the present study, we aim to evaluate the association of coffee intake and the prevalence of newly diagnosed diabetes as well as diabetic pathogenesis in a large high-risk diabetic Chinese Population from Shanghai High-risk Diabetic Screen project (SHiDS).

## Methods

### Study design and population

The study population was selected from the Shanghai High-Risk Diabetic Screen (SHiDS) project, a large clinic based screening project that was implemented since 2002. Details on the methodology have previously been reported [11]. In brief, the SHiDS project involves screening of individuals with known risk factors for diabetes. The inclusion criterion of SHiDS study is individuals with at least one of the known risk factors for diabetes including 1) family history of diabetes, 2) being overweight or obese, 3) previously identified impaired fasting glucose or impaired glucose tolerance, 4) history of gestational diabetes, 5) polycystic ovary syndrome, 6) hypertension, 7) dyslipidemia. Previously diagnosed diabetic patients were excluded from the study [11]. For the present study, a total of 1328 individuals over 18 years of age who have the information of coffee intake were enrolled in the study from 2012 to 2016. This study was approved by the Institutional Review Board of Shanghai JiaoTong University Affiliated Sixth People’s Hospital in accordance with the principles of the Helsinki Declaration. Informed consent was obtained from each subject before the survey.

### Data collection

Participants arrived at the hospital at 7: 30 AM after an 8 h overnight fasting before the five-point 75 g oral glucose tolerance test (OGTT). Each participant underwent a physical examination including measurement of height, weight, waist circumference and blood pressure. Body mass index (BMI) was calculated as weight divided by height squared (kg/m^2^). A standard questionnaire was conducted by trained research staff. Data on the current lifestyle, coffee intake, tea intake, smoking habit, alcohol intake, physical activity, medical record, diabetes family history (first degree relatives only) and educational status were collected for each participant. Venous blood samples were collected at 0, 30, 60, 120 and 180 min during a standard 75 g-OGTT, and plasma glucose levels were measured using a glucose oxidase method. Glycosylated hemoglobin A1c (HbA1c) was measured by high performance liquid chromatography. Glycated albumin (GA) was measured by the liquid enzymatic assay. Serum insulin levels were measured using a chemical luminescence method.

Diabetes was diagnosed according to the standard criteria by the World Health Organization (WHO) in 1999 [fasting plasma glucose (FPG) ≥7.0 mmol/L or 2-h postprandial plasma glucose (PG2h) ≥11.1 mmol/L]. Hypertension and dislipidemia were defined as self-reported with a validated history diagnosed by doctors. Insulin resistance was assessed by homeostatic model assessment of insulin resistance (HOMA-IR) value [12]. Pancreatic beta cell function was evaluated by homeostatic model assessment of β-cell function (HOMA-β) value [13], Stumvoll first phase and second phase indexes, respectively [14].

HOMA-IR = fasting serum insulin (FIN) (mU/L) × FPG (mmol/L) / 22.5 [12]

HOMA-β = 20 × FIN (mU/L) / [FPG (mmol/L) - 3.5] [13]

Stumvoll1st phase = 2503 + 38.856 × FIN (mU/L) - 126.5 × PG2h (mmol/l) + 5.724 × 2-h postprandial serum insulin (IN2h) (mU/L) - 239.3 × FPG (mmol/l) [14].

Stumvoll 2nd phase = 393 + 6.978 × FIN (mU/L) - 40.72 × PG2h (mmol/l) + 1.878 × IN2h (mU/L) [14]

### Statistical analyses

Statistical analyses were performed using SPSS 20.0 (SPSS, Inc., Chicago, IL). FPG, 30-min postprandial plasma glucose (PG30min), 1-h postprandial plasma glucose (PG1h), PG2h, 3-h postprandial plasma glucose (PG3h), FIN, 30-min postprandial serum insulin (IN30min), 1-h postprandial serum insulin (IN1h), IN2h, 3-h postprandial serum insulin (IN3h), HbA1c, GA and HOMA-IR were logarithmic transformed to obtain approximate normal distribution before statistical analysis. Participant characteristics were tested for differences across coffee intake categories. Variables with approximately normal distribution were presented as means ± standard deviation while those with skew distribution were shown as median [interquartile range (IQR)] and classified variable as frequencies and percentages. Continuous variables were compared by t-test while classified variable by chi-square test. The associations of coffee intake with different variables were analyzed by covariance. Logistic regression was used to model associations between coffee consumption and insulin secretion indexes, taking account of potential confounders. All reported *P* values were two-tailed and *P* < 0.05 were considered statistically significant.

## Result

### Clinical characteristics of the participants stratified by coffee consumption

The demographic and clinical characteristics of the participants are presented in Table 1. After statistical analysis by t-test, it showed that coffee consumed population was relatively younger and fatter than non-coffee participants (*P* for age < 0.001; *P* for BMI < 0.001; *P* for waist < 0.001; *P* for fat% = 0.004). Besides, coffee consumed group had higher education level (*P* <  0.001), current smoke percentage (*P* = 0.010), current alcohol intake percentage (*P* = 0.001), tea consumption percentage (*P* < 0.001) and higher percentage of diabetes family history (*P* = 0.041). The prevalence of hypertension and dyslipidemia were not different between these two groups.

**Table 1:** Clinical characteristics of the participants stratified by coffee consumption

	Non-coffee (*n* = 818)	Coffee (*n* = 510)	Total (*n* = 1328)	*P*
Male (n, %)	330 (40.34%)	210 (41.18%)	540 (40.66%)	0.763
Age, years (mean, SD)	52.80 (14.22)	48.34 (14.49)	51.08 (14.47)	< 0.001
Weight, kg (mean, SD)	65.07 (12.60)	69.99 (14.24)	66.95 (13.46)	< 0.001
BMI, kg/m^2^ (mean, SD)	23.96 (3.65)	25.18 (4.08)	24.43 (3.87)	< 0.001
Waist, cm (mean, SD)	86.46 (10.19)	88.79 (11.07)	87.35 (10.59)	< 0.001
Fat, % (mean, SD)	28.37 (7.70)	29.81 (8.25)	28.91 (7.94)	0.004
SBP, mmHg (mean, SD)	130.39 (18.17)	130.23 (17.41)	130.33 (17.88)	0.880
DBP, mmHg (mean, SD)	79.02 (10.93)	79.20 (11.33)	79.09 (11.09)	0.776
Diabetic family history (n, %)	351 (44.94%)	249 (50.82%)	1271 (47.21%)	0.041
Hypertension (n, %)	279 (34.15%)	160 (31.37%)	1327 (33.08%)	0.296
Dislipidemia (n, %)	249 (30.48%)	168 (33.01%)	1326 (31.45%)	0.335
Higher Education (n, %)	370 (45.23%)	312 (61.18%)	1328 (51.36%)	< 0.001
Current smoke (n, %)	104 (12.73%)	91 (17.88%)	1326 (14.71%)	0.010
Current alcohol intake (n, %)	115/703 (14.06%)	107/402 (21.02%)	222 (16.73%)	0.001
Tea consumption (n, %)	293 (35.91%)	321 (62.94%)	1326 (46.30%)	< 0.001
Frequent physical activity (n, %)	629 (76.99%)	385 (75.49%)	1327 (76.41%)	0.532
FPG, mmol/L (median, IQR)	6.15 (5.46–7.06)	6.04 (5.36–7.14)	6.11 (5.42–7.08)	0.259
PG30min, mmol/L (median, IQR)	10.67 (9.09–12.38)	10.47 (9.05–12.27)	10.60 (9.07–12.35)	0.188
PG1h, mmol/L (median, IQR)	12.29 (9.79–15.25)	12.19 (9.43–14.97)	12.25 (9.68–15.14)	0.148
PG2h, mmol/L (median, IQR)	10.02 (7.36–14.31)	9.53 (7.09–13.47)	9.85 (7.28–14.06)	0.024
PG3h, mmol/L (median, IQR)	6.69 (4.84–9.62)	6.30 (4.55–8.97)	6.45 (4.74–9.35)	0.016
FIN, uIU/mL (median, IQR)	8.43 (5.75–12.61)	10.10 (6.94–16.22)	9.08 (6.02–13.81)	< 0.001
IN30min, uIU/mL (median, IQR)	44.05 (27.52–69.97)	58.09 (34.50–93.70)	48.21 (28.82–80.23)	< 0.001
IN1h, uIU/mL (median, IQR)	64.58 (40.18–101.45)	77.03 (50.30–121.83)	69.20 (44.25–107.50)	< 0.001
IN2h, uIU/mL (median, IQR)	69.91 (45.83–115.70)	81.54 (51.53–128.38)	74.75 (48.05–119.05)	0.002
IN3h, uIU/mL (median, IQR)	32.22 (17.34–57.65)	34.29 (18.23–62.04)	32.75 (17.64–58.38)	0.237
HbA1c, % (median, IQR)	6.00 (5.60–6.50)	5.90 (5.50–6.60)	5.90 (5.60–6.50)	0.855
GA, % (median, IQR)	14.30 (12.80–16.60)	13.85 (12.40–16.70)	14.10 (12.70–16.68)	0.263
Newly diagnosed diabetes (n, %)	381 (46.58%)	213 (41.76%)	594 (44.73%)	0.037
Impaired glucose regulation (n, %)	237 (28.97%)	148 (29.02%)	385 (28.99%)	0.241
HOMA-β (mean, SD)	84.30 (89.77)	104.65 (103.29)	92.11 (95.66)	< 0.001
HOMA-IR (median, IQR)	2.37 (1.49–3.71)	2.80 (1.75–4.61)	2.52 (1.60–4.04)	< 0.001
Stumvoll first phase (mean, SD)	459.97 (1096.52)	717.67 (1060.32)	558.85 (1089.60)	< 0.001
Stumvoll second phase (mean, SD)	179.32 (248.52)	242.04 (246.55)	203.33 (249.54)	< 0.001

Continuous variables were compared by t-test while classified variable by chi-square test

Variables with approximately normal distribution were presented as means ± standard deviation while those with skew distribution were shown as median (inter quartile range) and classified variable as frequencies and percentages

Abbreviations: *SD* standard deviation, *IQR* inter quartile range, *BMI* body mass index, *SBP* systolic pressure, *DBP* diastolic pressure, *FPG* fasting plasma glucose, *PG30min* 30-min postprandial plasma glucose, *PG1h* 1-h postprandial plasma glucose, *PG2h* 2-h postprandial plasma glucose, *PG3h* 3-h postprandial plasma glucose, *HbA1c* glycosylated hemoglobin A1c, *GA* glycated albumin, *FIN* fasting serum insulin, *IN30min* 30-min postprandial serum insulin, *IN1h* 1-h postprandial serum insulin, *IN2h* 2-h postprandial serum insulin, *IN3h* 3-h postprandial serum insulin, *HOMA-IR* homeostatic model assessment of insulin resistance, HOMA-β, homeostatic model assessment of β-cell function

In terms of glucose metabolism, the coffee intake group had lower 2-h and 3-h plasma glucose levels during OGTT (*P* for PG2h = 0.024; *P* for PG3h = 0.016). However, the plasma glucose levels at fasting, 30-min, 1-h were not different between these two populations, so as the HbA1c level and GA level. According to the chi-square test, coffee intake group had lower prevalence of newly diagnosed diabetes (*P* = 0.037).

In terms of insulin levels, coffee consumption group had higher insulin levels at fasting, 30-min, 1-h and 2-h during OGTT (*P* for FIN < 0.001; *P* for IN30min < 0.001; *P* for IN1h < 0.001; *P* for IN2h = 0.002), but serum insulin level at 3-h was not different from the non-coffee intake group. Pancreatic beta cell function and insulin resistance were further estimated by HOMA and Stumvoll indexes. It implied that coffee intake individuals had higher insulin secretion level (*P* for HOMA-β < 0.001; *P* for Stumvoll first phase < 0.001; *P* for Stumvoll second phase < 0.001) and lower insulin sensitivity (*P* for HOMA-IR < 0.001).

### Coffee consumption and glucose metabolism, insulin secretion and insulin sensitivity

The relationships of coffee intake and glucose metabolism, insulin secretion and insulin sensitivity were further analyzed by different adjusted models (Table 2). First of all, coffee consumption group had lower plasma glucose levels at 2-h and 3-h and higher insulin levels at fasting, 30-min and 1-h during OGTT after adjustment with age, fat%, BMI, waist, tea intake, smoking habit, alcohol intake, diabetes family history and educational status (*P* for PG2h = 0.002; *P* for PG3h = 0.010; *P* for FIN = 0.010; *P* for IN30min = 0.001; *P* for IN1h = 0.002). Nevertheless, the difference of the prevalence of newly diagnosed diabetes between these two groups was not significant after adjusted with age (*P* = 0.718). Secondly, a relatively strong association was found between coffee consumption and insulin secretion after adjusted with variety confounders mentioned above that might interfere with the relationship of coffee intake and pancreatic beta cell function. Both HOMA-β and Stumvoll formula indexes were positively related to coffee consumption (*P* for HOMA-β = 0.033; *P* for Stumvoll first phase = 0.003; *P* for Stumvoll second phase = 0.001). However, insulin sensitivity index was not statistically different between coffee consumed population and non-coffee consumed population after adjustment.

**Table 2:** Adjusted Mean (SD) values for metabolic measures according to the relationship with coffee consumption

lgPG2h				lgPG3h			
	Non-coffee	Coffee	P*		Non-coffee	Coffee	P*
Model 1	1.01 ± 0.18	0.98 ± 0.19	0.477	Model 1	0.84 ± 0.21	0.81 ± 0.21	0.122
Model 2	1.01 ± 0.18	0.99 ± 0.19	0.020	Model 2	0.85 ± 0.21	0.82 ± 0.21	0.021
Model 3	1.02 ± 0.18	0.99 ± 0.19	0.002	Model 3	0.85 ± 0.21	0.82 ± 0.21	0.010
lgFIN				lgIN30min			
	Non-coffee	Coffee	P*		Non-coffee	Coffee	P*
Model 1	0.92 ± 0.29	1.01 ± 0.29	< 0.001	Model 1	1.64 ± 0.34	1.74 ± 0.36	< 0.001
Model 2	0.92 ± 0.30	1.01 ± 0.29	0.012	Model 2	1.64 ± 0.34	1.75 ± 0.35	0.002
Model 3	0.92 ± 0.29	1.01 ± 0.29	0.010	Model 3	1.64 ± 0.34	1.74 ± 0.35	0.001
lgIN1h				lgIN2h			
	Non-coffee	Coffee	P*		Non-coffee	Coffee	P*
Model 1	1.80 ± 0.31	1.87 ± 0.32	< 0.001	Model 1	1.85 ± 0.31	1.90 ± 0.31	0.004
Model 2	1.79 ± 0.30	1.87 ± 0.31	0.014	Model 2	1.85 ± 0.31	1.90 ± 0.30	0.287
Model 3	1.79 ± 0.30	1.87 ± 0.31	0.002	Model 3	1.85 ± 0.31	1.90 ± 0.30	0.120
lgHOMA-IR				HOMA-β			
	Non-coffee	Coffee	P*		Non-coffee	Coffee	P*
Model 1	0.37 ± 0.32	0.44 ± 0.33	< 0.001	Model 1	84.30 ± 89.77	104.65 ± 103.29	0.025
Model 2	0.37 ± 0.33	0.45 ± 0.32	0.053	Model 2	80.59 ± 85.37	104.70 ± 96.93	0.090
Model 3	0.37 ± 0.32	0.45 ± 0.32	0.085	Model 3	78.06 ± 70.61	99.44 ± 74.48	0.033
Stumvoll first phase				Stumvoll second phase			
	Non-coffee	Coffee	P*		Non-coffee	Coffee	P*
Model 1	459.97 ± 1096.52	717.67 ± 1060.32	0.005	Model 1	179.32 ± 248.52	242.04 ± 246.55	0.002
Model 2	415.95 ± 1102.94	681.45 ± 1041.09	0.045	Model 2	170.08 ± 249.30	236.52 ± 236.91	0.020
Model 3	392.53 ± 1101.89	659.70 ± 1029.83	0.003	Model 3	166.15 ± 249.01	232.98 ± 233.96	0.001

Values presented as Mean ± SD. PG2h, PG3h, FIN, IN30min, IN1h, IN2h and HOMA-IR were logarithmic transformed

*P-value for the test of any association between coffee consumption and the outcome of interest

Model 1: adjusted for age

Model 2: Model 1 + BMI, Fat%, Waist

Model 3: Model 2 + education status, smoke status, alcohol intake, tea consumption, diabetes family history

Abbreviations: *SD* standard deviation, *BMI* body mass index, *PG2h* 2-h postprandial plasma glucose, *PG3h* 3-h postprandial plasma glucose, *FIN* fasting serum insulin, *IN30min* 30-min postprandial serum insulin, *IN1h* 1-h postprandial serum insulin, *IN2h* 2-h postprandial serum insulin, *HOMA-IR* homeostatic model assessment of insulin resistance, *HOMA-β* homeostatic model assessment of β-cell function

### Logistic regression analysis about the relationship of insulin secretion and coffee consumption

Logistic regression was conducted to further analysis the relationship of coffee intake and insulin secretion (Table 3). Insulin secretion indexes were expressed in quartiles and were taken as dependent variable. Coffee intake and the other parameters (age, gender distribution, BMI, fat%, waist, education status, smoke status, alcohol intake, tea consumption, diabetes family history) were taken as independent variables. It showed that coffee consumption was significantly and positively associated with insulin secretion. After adjusted with the confounders (model 1, model 2 and model 3), Logistic regression analysis further confirmed that coffee consumers had higher levels of HOMA-β and Stumvoll insulin secretion indexes {model 3: odds ratio (OR) [95% confidence interval (CI)] for HOMA-β = 2.262 (1.451–3.527); OR (95% CI) for Stumvoll first phase = 2.041 (1.331–3.131); OR (95% CI) for Stumvoll second phase = 1.890 (1.243–2.874)}. Thus, participant consumed coffee might have better insulin secretion function, including the basic insulin secretion and the two phases of insulin secretion during OGTT.

**Table 3:** Odds ratios (95% CI) for the association between coffee consumption and insulin secretion indexes

	HOMA-β (the highest quartiles)		
coffee consumption	OR	95% CI	P*
Model 1	2.240	1.598–3.140	< 0.001
Model 2	2.048	1.359–3.086	0.001
Model 3	2.262	1.451–3.527	< 0.001
	Stumvoll first phase (the highest quartiles)		
coffee consumption	OR	95% CI	P*
Model 1	1.818	1.297–2.549	0.001
Model 2	1.807	1.222–2.672	0.003
Model 3	2.041	1.331–3.131	0.001
	Stumvoll second phase (the highest quartiles)		
coffee consumption	OR	95% CI	P*
Model 1	1.730	1.238–2.419	0.001
Model 2	1.762	1.196–2.595	0.004
Model 3	1.890	1.243–2.874	0.003

*P-value for the test of any association between coffee consumption and the outcome of interest

Model 1: adjusted for age, gender distribution

Model 2: Model 1 + BMI, Fat%, Waist

Model 3: Model 2 + education status, smoke status, alcohol intake, tea consumption, diabetes family history

## Discussion

As far as we know, the present study was the first investigation about relationship between coffee intake and diabetes as well as its pathogenesis in a large high-risk diabetic Chinese population. It showed that coffee intake was positively associated with fasting and the first hour insulin levels and inversely associated with second and third hour plasma glucose levels during the OGTT. In addition, Logistic analysis revealed that coffee consumption was independently and positively related to a series of insulin secretion indexes, including HOMA-β, Stumvoll first and second phase insulin secretion indexes. The HOMA calculation is derived from a computer solved model that assumes relationships between fasting plasma glucose and insulin concentration [13]. HOMA-β evaluated the basic insulin secretion function [15]. Stumvoll 1st and 2nd phase insulin secretion indexes are simple demographic parameters and they can evaluate insulin release accuracy [16]. The significance of the first phase insulin secretion might reflect the existence of a compartment of readily releasable insulin within the beta cell or a transientrise and fall of a metabolic signal for insulin secretion [17, 18]. The second phase of insulin secretion is directly related to the level of glucose elevation [18]. Thus, the present study implied that coffee consumers not only had better basal pancreatic beta cell function, but also had superior postprandial beta cell function.

Our findings were consistent with some previous investigations. The results from Agardh et al. showed that in those with type 2 diabetes high coffee consumption was inversely associated with decreased beta cell function which was evaluated by HOMA-β [8]. It suggested an effect of coffee on beta-cell function and there was a tendency of improved insulin response in those with high coffee consumption [8]. The results from Wu TY et al. provided support for the potential benefit of chronic coffee consumption on insulin secretion and possibly diabetes [19, 20]. However, there are some inconsistencies in the effect of coffee on insulin secretion. In a cross-sectional analysis of Japanese population, higher coffee consumption was not associated with insulin secretion, as evaluated through the HOMA [21]. Another cross-sectional study from the Uppsala Longitudinal Study of Adult Men showed that there was no association between coffee consumption and early insulin response during an OGTT [9]. The differences in race and population selection criteria might cause different results of relationship between coffee intake and insulin secretion.

The mechanisms that coffee may have beneficial effects on pancreatic beta cell function are still unclear. Coffee contains a lot of components. Caffeine is the major one which is already known to enhance insulin secretion [8, 22]. It has been proved that insulin concentration tended to be higher in the first 30 min after caffeinated coffee consumption compared with that of decaffeinated coffee or water [23]. Another study revealed that insulin was significantly higher after caffeine coffee than after water during the first hour of the OGTT [24]. Other major components of coffee including magnesium, chlorogenic acid and various other micronutrients could also be involved in insulin secretion [25]. Chlorogenic acid is another major ingredient in coffee. There is evidence that chlorogenic acid might stimulate glucagon-like peptide 1 (GLP-1) production which is one of the gastrointestinal hormones and is known to have an effect on beta cell function that amplifies glucose-dependent insulin secretion [23, 26]. On the other hand, magnesium has also been shown to improve β-cell function. Previous study found that increase in erythrocyte magnesium significantly and positively correlated with the increase in both insulin secretion and action [27]. Thus, more and deeper investigations are necessary to reveal the mechanisms that coffee is benefit for insulin secretion and glucose homeostasis.

There are some strengths of our study. First of all, each diabetic patient was newly diagnosed by astandard 75 g-OGTT. Thus, the effect of anti-diabetic medication on the relationship of coffee intake and hyperglycemia can be excluded. We also tested the 5-point blood glucose and insulin levels during the OGTT, which made it possible for us to entirely investigate the dynamic fluctuations in insulin secretion after glucose load. Secondly, to our knowledge, there were few studies on coffee intake and hyperglycemia in Chinese population. The population of China accounts for one fifth of the world’s population and China is becoming an emerging coffee consumer country. According to the 2017–2021 China coffee industry development prospect forecast and investment analysis report, coffee consumption is growing by 15 to 20% a year in China [28]. Thus, it is especially important to evaluate the relationship of coffee intake with glucose metabolism in Chinese population. Our study extended the apparent protective effect of coffee on insulin secretion and glucose metabolism.

Except for the major results of the present study, we also found that coffee consumed group had higher current smoke percentage, current alcohol intake percentage and tea consumption percentage. These results were consistent with the previous studies [8, 19, 25, 29]. It is well-known that alcohol, tobacco, tea and coffee can cause varying degrees of addiction or dependence [30–32]. Our findings implied that there might be some concentration effect on dependency or addictive substances, i.e. coffee, tea, alcohol and tobacco, which needs further study.

## Conclusions

In the present study, we provide solid and strong evidence that coffee intake is independently and positively related to pancreatic beta cell function in a large high-risk diabetic Chinese population. Habitual coffee consumption population might have better pancreatic beta cell function in Chinese population.

## Abbreviations

BMI: Body mass index
CI: Confidence interval
DBP: Diastolic pressure
FIN: Fasting serum insulin
FPG: Fasting plasma glucose
GA: Glycated albumin
GLP-1: Glucagon-like peptide 1
HbA1c: Glycosylated hemoglobin A1c
HOMA-IR: Homeostatic model assessment of insulin resistance
HOMA-β: Homeostatic model assessment of β-cell function
IN1h: 1-h postprandial serum insulin
IN2h: 2-h postprandial serum insulin
IN30min: 30-min postprandial serum insulin
IN3h: 3-h postprandial serum insulin
IQR: Inter quartile range
OGTT: Oral glucose tolerance test
OR: odds ratio
PG1h: 1-h postprandial plasma glucose
PG2h: 2-h postprandial plasma glucose
PG30min: 30-min postprandial plasma glucose
PG3h: 3-h postprandial plasma glucose
SBP: Systolic pressure
SD: Standard deviation
SHiDS: Shanghai High-Risk Diabetic Screen
WHO: World Health Organization
